# Essential oil mixture on rumen fermentation and microbial community – an *in vitro* study

**DOI:** 10.5713/ajas.18.0652

**Published:** 2018-11-27

**Authors:** Hanbeen Kim, Eunsang Jung, Hyo Gun Lee, Byeongwoo Kim, Seongkeun Cho, Seyoung Lee, Inhyuk Kwon, Jakyeom Seo

**Affiliations:** 1 Department of Animal Science, Life and Industry Convergence Research Institute, Pusan National University, Miryang 50463, Korea; 2 Department of Bioenvironmental Energy, Life and Industry Convergence Research Institute, Pusan National University, Miryang 50463, Korea; 3 Division of Animal Husbandry, Yonam College, Cheonan 31005, Korea; 4 EASY BIO, Inc., Seoul 06253, Korea

**Keywords:** Feed Additive, *In vitro* Degradability, Microbial Abundance

## Abstract

**Objective:**

The objective of this study was to investigate the effects of essential oil mixture (EOM) supplementation on rumen fermentation characteristics and microbial changes in an *in vitro*.

**Methods:**

Three experimental treatments were used: control (CON, no additive), EOM 0.1 (supplementation of 1 g EOM/kg of substrate), and EOM 0.2 (supplementation of 2 g EOM/kg of substrate). An *in vitro* fermentation experiment was carried out using strained rumen fluid for 12 and 24 h incubation periods. At each time point, *in vitro* dry matter digestibility (IVDMD), neutral detergent fiber digestibility (IVNDFD), pH, ammonia nitrogen (NH_3_-N), and volatile fatty acid (VFA) concentrations, and relative microbial diversity were estimated.

**Results:**

After 24 h incubation, treatments involving EOM supplementation led to significantly higher IVDMD (treatments and quadratic effect; p = 0.019 and 0.008) and IVNDFD (linear effect; p = 0.068) than did the CON treatment. The EOM 0.2 supplementation group had the highest NH_3_-N concentration (treatments; p = 0.032). Both EOM supplementations did not affect total VFA concentration and the proportion of individual VFAs; however, total VFA tended to increase in EOM supplementation groups, after 12 h incubation (linear; p = 0.071). Relative protozoa abundance significantly increased following EOM supplementation (treatments, p<0.001). *Selenomonas ruminantium* and *Ruminococcus albus* (treatments; p<0.001 and p = 0.005), abundance was higher in the EOM 0.1 treatment group than in CON. The abundance of *Butyrivibrio fibrisolvens*, fungi and *Ruminococcus flavefaciens* (treatments; p< 0.001, p<0.001, and p = 0.005) was higher following EOM 0.2 treatment.

**Conclusion:**

The addition of newly developed EOM increased IVDMD, IVNDFD, and tended to increase total VFA indicating that it may be used as a feed additive to improve rumen fermentation by modulating rumen microbial communities. Further studies would be required to investigate the detailed metabolic mechanism underlying the effects of EOM supplementation.

## INTRODUCTION

Numerous ruminant nutritionists have tried to develop natural alternatives for antibiotics since the prohibition of their use in livestock industry in the European Union owing to the risk of antibiotic residues in animal products [1] and appearance of multi-drug resistant bacteria that might have a negative impact on human health [2]. Essential oils (EO) are secondary metabolites from plants that function in defense against herbivores and invading microbes and have been known to possess antimicrobial properties [3,4]. In addition, plant extracts are categorized in generally recognized as safe for human consumption; therefore, numerous studies have evaluated the efficacy of EO and essential oil compounds (EOC) with the aim of replacing antibiotic rumen modulators such as monensin, using an *in vitro* batch culture systems [2,5–7] and continuous culture systems [8–10].

In the previous studies, the separate use of individual EO (eugenol, thymol, and cinnamaldehyde) in an *in vitro* system has shown various effects, but results were inconsistent with respect to the modulation of ruminal fermentation [2,8,9,11]. Therefore, the use of essential oil mixtures (EOM) containing various EO has been widely assessed for the modulation of rumen fermentation, and the type and quantity of the EO in an EOM are the most important factors that determine their capability for modulating rumen fermentation [5]. Novatan (Techna, Couëron, France), a commercial EOM, has been developed for reducing excessive protein degradation in the rumen through the synergetic activity of EO (eugenol, thymol, and cinnamaldehyde) and microminerals (Na, Zn, and Mn) [12].

The aim of this study was thus to investigate the effects of dietary supplementation of a newly developed commercial EOM containing eugenol, thymol, and cinnamaldehyde, on rumen fermentation characteristics and changes in microbial diversity using an *in vitro* batch culture system.

## MATERIALS AND METHODS

Animal use and the protocols for this experiment were reviewed and approved by the Animal Research Ethics Committee of Pusan National University.

### Preparation of an experimental substrate

A commercial concentrate mix for fattening beef was purchased from a feed company (Easybio, Inc., Seoul, Korea). The detailed chemical composition was as follows: dry matter (DM), 900 g/kg; crude protein, 255 g/kg; neutral detergent fiber (NDF), 246 g/kg; acid detergent fiber (ADF), 116 g/kg; lignin, 35.3 g/kg; ether extracts, 48.1 g/kg; ash, 87.3 g/kg; Ca, 4.3 g/kg; P, 7.8 g/kg. The experimental substrates were dried at 60°C for 96 h, and ground in a cyclone mill (Foss Tecator Cyclotec 1093, Foss, Hillerød, Denmark) fitted with a 1 mm screen, for later use. A commercial EOM (NOVATAN, Techna, France) was used in this study as a feed additive. According to the manufacturer, the main constituents in the EOM were cinnamaldehyde, thymol, and eugenol.

### Experimental treatments

A complete randomized block design was used for the experiment, with treatment as the main effect. Three experimental treatments were used, i.e., control (CON, no additive), EOM 0.1 (1 g EOM/kg of experimental substrate), EOM 0.2 (2 g EOM/kg of experimental substrate). The amount of EOM added was determined based on the manufacturer’s recommendation.

### *In vitro* incubation

*In vitro* batch culture fermentation was carried out using rumen fluid, which was collected before the morning feeding from two cannulated Korean native cattle heifers (body weight 450±30 kg) at Center for Animal Science Research, Gyeongsang National University, Korea. Animals were fed a diet consisting of 600 g/kg timothy hay and 400 g/kg of a commercial concentrate mix. The rumen contents were mixed, transferred into a thermos bottle, and immediately transported to the laboratory. Rumen contents were strained through 4 layers of cheesecloth and mixed with 3× volumes of *in vitro* rumen buffer solution [13] under strictly anaerobic conditions. Approximately 0.9 g DM of the ground experimental substrate and a corresponding quantity of EOM for each treatment were moved into pre-weighed filter bags (F57, Ankom Technology, Macedon, NY, USA). All bags were heat-sealed and transferred into empty 125 mL serum bottles, to which 50 mL of rumen fluid and buffer mixture were transferred accompanied by continuously flushing with O_2_-free CO_2_ gas. The bottles were sealed with butyl rubber stoppers and aluminum caps and incubated on a rotary shaker (JSSI-300T, JS Research Inc., Gongju, Korea) at 125 rpm, for 12 and 24 h at 39°C. Three replicates were prepared for each treatment and each incubation time. After each incubation period, the three replicates of each treatment were removed from the incubator, and total gas production was measured using a pressure transducer (Sun Bee Instrument Inc., Seoul, Korea) as described by Theodorou et al [14]. Subsequently, the bottles were immediately placed on ice and the caps were opened to stop the fermentation; the filter bags were removed from bottles and rinsed with flowing water until the water ran clear. The pH of the culture fluid was measured using a pH meter (FP20, Mettler Toledo, Columbus, OH, USA). Sample fluid (5 mL) was stored at −80°C until rumen microbial population analysis. The remaining culture fluid was then centrifuged at 15,000×*g* for 10 min at 4°C and stored at −20°C, until the analysis of volatile fatty acids (VFA) and ammonia nitrogen (NH_3_-N) concentrations. The washed bags were dried at 55°C for 48 h and weighed to measure *in vitro* DM degradability (IVDMD). The NDF content was assessed for the weighed bags using a modified version of the micro-NDF method proposed by Pell and Schofield [15] to evaluate *in vitro* NDF degradability (IVNDFD).

### Total DNA extraction

Collected rumen fluid samples were thawed in a refrigerator at 4°C, and centrifuged at 16,000×*g* and 4°C for 5 min. The supernatant was discarded, and the remainder was resuspended in 800 μL of 1× phosphate-buffered saline solution to wash the pellet. This was recentrifuged (16,000×*g*, 4°C, 5 min) and the supernatant was discarded. Total DNA was extracted from the washed pellet using a NucleoSpin DNA Stool kit (MACHEREY-NAGEL GmbH Co. KG, Düren, Germany), according to the manufacturer’s instructions. The concentration and purity of total DNA were measured using a NanoDrop (ND-1000, Thermo Fisher, Waltham, MA, USA).

### Conventional polymerase chain reaction for validation of primer and real-time polymerase chain reaction

The information on primer sequences of rumen microbes was collected from previous studies [16,17] and presented in Table 1. Conventional polymerase chain reaction (PCR) was performed to validate the specificity of the primer in 50 μL reaction volumes using AccuPower PCR PreMix K-2013 (Bioneer, Daejeon, Korea), by adding 2 μL each of forward and reverse primers, 2 μL of genomic DNA and 44 μL of PCR grade water. Reactions were performed using a C1000 Thermal cycler (Bio-Rad, Hercules, CA, USA) under the following conditions: initialization for one cycle at 95°C for 10 min; 35 cycles each for denaturation at 95°C for 30 s, annealing at 60°C for 30 s, and elongation at 72°C for 30 s; and final elongation at 72°C for 5 min. The PCR products were analyzed by electrophoresis using 1.5% agarose gel impregnated with Staining STAR (DYNEBIO INC, Seongnam, Korea), to confirm a single specific band and the absence of primer dimer products.

Real-time quantitative PCR assays were performed on a CFX 96 Touch system (Bio-Rad Laboratories, lnc. USA). Reactions were performed in triplicate with reaction volumes of 20 μL using optical reaction plates sealed with optical adhesive film. Each reaction mixture contained 0.5 μL 10 mM dNTP Mix (BioFACT, Daejeon, Korea), 2 μL 10× buffer (BioFACT, Korea), 1 μL 40 ng genomic DNA, 1 μL forward primer (10 pmol), 1 μL reverse primer (10 pmol), 0.1 μL Taq polymerase (BioFACT, Korea), 1 μL Evagreen (SolGent Co., Ltd., Daejeon, Korea) and 13.4 μL PCR grade water. Real-time PCR was carried out according to the manufacturer’s instructions, as follows: initialization for one cycle at 95°C for 10 min; 40 cycles each for denaturation at 95°C for 30 s, annealing at 60°C for 30 s and elongation at 72°C for 30 s; and final elongation at 72°C for 5 min. Fluorescence was recorded at the end of each denaturation and extension step, and specificity of amplicon was confirmed via dissociation curve analysis of PCR end products by increasing the temperature at a rate of 1°C after every 30 s, from 60°C to 95°C. The threshold cycle (*C*t) was analyzed and transformed using a standard curve with CFX manager software (Bio-Rad, USA).

### Relative genomic DNA abundance of genes

To evaluate the efficiency (E) of amplification of each primer set, a 6-point standard curve and a nontemplate control (NTC) were run for each sample to test the relative expression levels. The standard curve was generated using genomic DNA (40 μg/reaction mixture), which was serially diluted. Amplification efficiency was calculated from the slope of the standard curve generated by plotting the threshold cycle (*C*t) against logarithmic values of different DNA concentrations using the equation, E = 10^−1/slope^. The gene ‘general bacteria’ was used as a reference gene. Relative quantification of microbes was carried out using the 2^−ΔΔCt^ method as follows [18,19]:

$$\begin{array}{l} \text{Relative quantification}\\ =2^{-[(C\text{t target gene}-C\text{t reference gene})\;\text{treatment group}-(C\text{t target gene}-C\text{t reference gene})\;\text{control group}]} \end{array}$$

### Statistical analysis

Statistical analysis of the data was performed using the MIXED procedure of SAS 9.3 (SAS institute Inc., NC, USA). The fixed effect in this model was a feed treatment. Orthogonal contrast was used to analyze linear and quadratic effect when the level of EOM supplementation increased. Differences among treatments were compared using Tukey’s range test if a significant effect was observed. Statistical significance was declared at p<0.05 and a trend was speculated at 0.05≤p<0.10.

## RESULTS

The effects of EOM on rumen fermentation characteristics are presented in Table 2. After incubating for 12 h, there was no significant difference in IVDMD, IVNDFD, pH, and NH_3_-N concentration, among treatments. After incubating for 24 h, The IVDMD was significantly changed in a quadratic manner (p = 0.008), and the IVNDFD tended to increase linearly when the EOM was supplemented (p = 0.068). The pH was quadratically changed in when EOM was increasingly added (p = 0.032). No significant effect on NH_3_-N concentrations was detected at 12 h incubation but, the highest NH_3_-N concentration was observed in the group of EOM 0.2 at 24 h fermentation (p = 0.032 for Trt, and p = 0.013 for linear).

Dietary addition of EOM tended to increase the total VFA concentration linearly after 12 h incubation (p = 0.071). There was no significant difference in total VFA concentration among treatments after 24 h incubation. Significant changes in the proportion of each VFA, and acetate to propionate ratio was not observed in any incubation times.

The EOM 0.1 treatment changed the fold change of *Selenomonas ruminantium* (p<0.001) *Fibrobacter succinogenes* (p = 0.047), and *Ruminococcus albus* (p = 0.005) in a quadratic manner (Table 3). EOM 0.2 treatment increased fold change of ciliate protozoa (p<0.001), *Butyrivibrio fibrisolvens* (treatment, linear, and quadratic effect: p<0.001, 0.002, and 0.007, respectively), fungi (treatment and linear effect: p = 0.001 and <0.001, respectively), and *Ruminococcus flavefaciens* (treatment, linear, and quadratic effect: p<0.001, <0.0001, and, 0.002, respectively). *Prevotella ruminicola* abundance tended to increase in a linear manner by EOM supplementation (p = 0.080).

## DISCUSSION

The EO supplementation in ruminant diets can change rumen microbial activity and fermentation characteristics because of their antimicrobial activity. Dorman and Deans [3] also reported that the inconsistent rumen fermentation results following the dietary addition of EO might be attributed to the type and structural configuration of EO when different kinds of EO are administrated together. Therefore, numerous researchers have been developing new EOM candidates for useful modulation of rumen environment. In this study, newly developed EOM containing eugenol, thymol, and cinnamaldehyde was evaluated for efficacy to improve rumen fermentation pattern by *in vitro* batch culture.

Results from this study revealed that appropriate dosage of EOM improves rumen degradability and microbial activity. Cinnamaldehyde consisting EOM has been known to bind to feed proteins and inhibit the amino acid decarboxylase in *Enterobacter aerogenes* by disturbing energy metabolism of bacterial cells [21]. However, NH_3_-N significantly increased in response to supplementation of the high dose of EOM. Ammonia nitrogen is an end product generated by microbial fermentation of protein sources in the feed. In the previous studies, the NH_3_-N was not affected by the addition of BEO [9,22] and, Benchaar et al [2] did not observe any significant changes in NH_3_-N concentrations when EOs or EOCs were added to substrates. Furthermore, Busquet et al [6] reported that the addition of eugenol and cinnamaldehyde to substrates at over doses of 300 mg/L, decreased NH_3_-N concentrations. Considering that our dosage level of EOM 0.2 was 30 mg/L, it is postulated that the dosage level of EOM in this study might be too low to modulate microbial degradative action on protein sources. Newbold et al [23] reported that defaunation of protozoa significantly decreased the rumen NH_3_-N concentration (−26%, p<0.001), indicating that, in this study, the increase of relative fold change of ciliate protozoa with an increase in the level of EOM supplementation might be linked with a higher concentration of NH_3_-N in EOM treatments than those in CON.

In present study, an increased IVDMD and IVNDFD were observed in groups treated with EOM after 24-h incubation (Table 3). Regarding with IVDMD, Benchaar et al [2] observed a significant decrease, when diets were supplemented with carvacrol (400 mg/L) and eugenol (800 mg/L) (p<0.05), but other EO (cinnamon leaf oil, clove leaf oil, sweet orange oil, oregano oil, and thyme oil) and EOC (cinnamaldehyde and thymol) had no effect on IVDMD in an *in vitro* batch culture system after 24 h incubation. Castillejos et al [9] reported that IVDMD was not changed by supplementation of EOM containing thymol, limonene, and guaiacol in an *in vitro* continuous culture system. Benchaar et al [2], Castillejos et al [7], and Fraser et al [10] observed a decrease in IVNDFD, and Benchaar et al [2] explained that fibrolytic bacteria might be sensitive to high concentrations of phenolic compounds in EO thereby fiber digestibility might be impaired by EO supplementation. *Fibrobacter succinogenes*, *Ruminococcus flavefaciens*, and *Ruminococcus albus* are the bacteria constituting 33.0%, 2.6%, and 46.0%, respectively, of the rumen fibrolytic bacteria [24,25]. Rumen ciliate protozoa have been known to have fibrolytic activity [23], and the fungi in the rumen have also been considered to produce fibrolytic enzymes [26]. In the present study, EOM 0.1 significantly increased the fold change of *Ruminococcus albus* (p = 0.005) and increased that of *Fibrobacter succinogenes* in a quadratic manner (p = 0.047). EOM 0.2 significantly increased the fold change of ciliate protozoa (p<0.001), *Butyrivibrio fibrisolvens* (p<0.001), fungi (p = 0.001), and *Ruminococcus flavefaciens* (p<0.001). Thus, the EOM used in this study may promote fibrolytic bacterial, protozoa, and fungal growth by unknown synergistic effect between chemical compounds and the main EO in the newly developed EOM, thereby improving ruminal degradability.

Regardless with increased digestibility, total VFA and those proportions after 24 h incubation were not changed by EOM supplementation. We expected that total VFA concentration would increase because the increase in nutrient digestibility and changes of central ruminal bacterial abundance were observed at 24 h incubation. Several studies reported that total VFA concentrations or their proportion were not affected by the supplementation of EO [26,27] irrespective of changes in ruminal nutrient digestibilities and the reason was not explained clearly as well. We postulated that the increase in protozoal abundance by EO addition might be linked with the increase in fiber digestibility without changes of VFA production because they engulf feed particles and ruminal bacteria thereby the bacterial fermentation contribution to VFA production would be impaired [22].

## CONCLUSION

The addition of newly developed EOM to an *in vitro* batch culture affected ruminal fermentation characteristics and microbial fold changes. An increased IVDMD and IVNDFD were observed when EOM was added. It was suggested that the newly developed EOM may be used as a feed additive to improve rumen fermentation. Further studies are required to investigate the detailed metabolic mechanism underlying the effects of EOM supplementation.

## Figures and Tables

**Table 1:** Polymerase chain reaction primers used in this study

Target species	Primer	Sequence (5′ → 3′)	Size (bp)	Efficiency1)	References
General bacteria	F	CGGCAACGAGCGCAACCC	130	1.974	[16]
	R	CCATTGTAGCACGTGTGTAGCC			
*Fibrobacter succinogenes*	F	GTTCGGAATTACTGGGCGTAAA	121	2.079	[16]
	R	CGCCTGCCCCTGAACTATC			
*Ruminococcus albus*	F	CCCTAAAAGCAGTCTTAGTTCG	176	1.828	[28]
	R	CCTCCTTGCGGTTAGAACA			
*Ruminococcus flavefaciens*	F	CGAACGGAGATAATTTGAGTTTACTTAGG	132	2.055	[16]
	R	CGGTCTCTGTATGTTATGAGGTATTACC			
Ciliate protozoa	F	GCTTTCGWTGGTAGTGTATT	234	2.005	[29]
	R	CTTGCCCTCYAATCGTWCT			
*Selenomonas ruminantium*	F	GGCGGGAAGGCAAGTCAGTC	83	1.974	[17]
	R	CCTCTCCTGCACTCAAGAAAGACAG			
*Butyrivibrio fibrisolvens*	F	ACCGCATAAGCGCACGGA	65	1.966	[30]
	R	CGGGTCCATCTTGTACCGATAAAT			
Fungi	F	GAGGAAGTAAAAGTCGTAACAAGGTTTC	120	2.000	[16]
	R	CAAATTCACAAAGGGTAGGATGATT			
*Prevotella ruminicola*	F	GCGAAAGTCGGATTAATGCTCTATG	78	2.079	[17]
	R	CCCATCCTATAGCGGTAAACCTTTG			

bp, base pair.

1) Efficiency is calculated as [10^−1/slope^].

**Table 2:** Fermentation characteristics after *in vitro* incubation of treatments using strained rumen fluid

Items	Treatments1)			SEM	p-value2)		
	
	CON	EOM 0.1	EOM 0.2		Trt	Lin	Quad
DM digestibility (%)							
12 h	37.1	36.7	38.6	1.95	0.607	0.452	0.532
24 h	47.5^b^	54.8^a^	49.5^ab^	1.85	0.019	0.307	0.008
NDF digestibility (% NDF)							
12 h	16.9	18.6	20.7	2.60	0.406	0.198	0.927
24 h	29.0	34.4	35.2	2.81	0.132	0.068	0.391
pH							
12 h	6.84	6.84	6.83	0.03	0.884	0.633	1.000
24 h	6.67	6.58	6.65	0.03	0.077	0.537	0.032
NH_3_-N (mg/100 mL)							
12 h	19.1	20.3	20.0	2.10	0.837	0.680	0.688
24 h	21.9^b^	24.4^ab^	30.9^a^	2.58	0.032	0.013	0.402
Total VFA (mM)							
12 h	55.0	59.8	62.8	3.58	0.167	0.071	0.789
24 h	83.4	82.4	84.2	4.04	0.907	0.857	0.700
Acetate (mmol/mol)							
12 h	65.3	65.4	64.9	0.76	0.785	0.610	0.660
24 h	59.5	58.8	59.6	0.62	0.387	0.893	0.188
Propionate (mmol/mol)							
12 h	23.3	22.8	23.2	0.54	0.611	0.850	0.349
24 h	28.2	28.6	28.4	0.40	0.585	0.606	0.385
Butyrate (mmol/mol)							
12 h	11.3	11.7	11.8	0.52	0.594	0.357	0.717
24 h	12.3	12.6	12.0	0.64	0.645	0.650	0.429
Acetate:propionate							
12 h	2.80	2.87	2.80	0.09	0.711	0.978	0.428
24 h	2.11	2.06	2.10	0.04	0.392	0.763	0.198

SEM, standard error of the mean; DM, dry matter; NDF, neutral detergent fiber analyzed with heat stable α-amylase; VFA, volatile fatty acids.

1) CON, an experimental substrate without supplementation; EOM 0.1, 1 g essential oil mixture (EOM)/kg of experimental substrate; EOM 0.2, 2 g EOM/kg of experimental substrate.

2) Trt, a treatment effect; Lin, a linear effect; Quad, a quadratic effect.

a,b Values in the same row with different letters differ significantly (p<0.05).

**Table 3:** Fold changes of rumen microbial populations after 24-h *in vitro* incubation of treatments using strained rumen fluid

Items	Treatments1)			SEM	p-value2)		
	
	CON	EOM 0.1	EOM 0.2		Trt	Linear	Quad
Ciliate protozoa	1.01^b^	1.09^b^	1.25^a^	0.052	<0.001	<0.001	0.399
*Selenomonas ruminantium*	1.04^b^	3.03^a^	1.32^b^	0.225	<0.001	0.194	<0.001
*Butyrivibrio fibrisolvens*	1.02^b^	0.96^b^	1.30^a^	0.080	<0.001	0.002	0.007
*Prevotella ruminicola*	1.00	0.96	0.92	0.051	0.206	0.080	0.953
*Fibrobacter succinogenes*	1.01	1.39	1.13	0.176	0.109	0.494	0.047
Fungi	1.02^b^	1.44^ab^	1.96^a^	0.214	0.001	<0.001	0.765
*Ruminococcus flavefaciens*	1.01^b^	1.01^b^	1.45^a^	0.076	<0.001	<0.001	0.002
*Ruminococcus albus*	1.08^b^	1.91^a^	1.11^b^	0.313	0.018	0.926	0.005

SEM, standard error of the mean.

1) CON, an experimental substrate without supplementation; EOM 0.1, 1 g essential oil mixture (EOM)/kg of experimental substrate; EOM 0.2, 2 g EOM/kg of experimental substrate.

2) Trt, a treatment effect; Lin, a linear effect; Quad, a quadratic effect.

ab Values in the same row with different letters differ significantly (p<0.05).

## References

[1] Russell JB, Houlihan AJ (2003). Ionophore resistance of ruminal bacteria and its potential impact on human health. FEMS Microbiol Rev.

[2] Benchaar C, Chaves AV, Fraser GR, Beauchemin KA, McAllister TA (2007). Effects of essential oils and their components on *in vitro* rumen microbial fermentation. Can J Anim Sci.

[3] Dorman HJ, Deans SG (2000). Antimicrobial agents from plants: antibacterial activity of plant volatile oils. J Appl Microbiol.

[4] Kim J, Marshall MR, Wei C-i (1995). Antibacterial activity of some essential oil components against five foodborne pathogens. J Agric Food Chem.

[5] Macheboeuf D, Morgavi DP, Papon Y, Mousset J-L, Arturo-Schaan M (2008). Dose–response effects of essential oils on *in vitro* fermentation activity of the rumen microbial population. Anim Feed Sci Technol.

[6] Busquet M, Calsamiglia S, Ferret A, Kamel C (2006). Plant extracts affect *in vitro* rumen microbial fermentation. J Dairy Sci.

[7] Castillejos L, Calsamiglia S, Ferret A (2006). Effect of essential oil active compounds on rumen microbial fermentation and nutrient flow in *in vitro* systems. J Dairy Sci.

[8] Busquet M, Calsamiglia S, Ferret A, Cardozo PW, Kamel C (2005). Effects of cinnamaldehyde and garlic oil on rumen microbial fermentation in a dual flow continuous culture. J Dairy Sci.

[9] Castillejos L, Calsamiglia S, Ferret A, Losa R (2005). Effects of a specific blend of essential oil compounds and the type of diet on rumen microbial fermentation and nutrient flow from a continuous culture system. Anim Feed Sci Technol.

[10] Fraser GR, Chaves A, Wang Y, McAllister T, Beauchemin K, Benchaar C (2007). Assessment of the effects of cinnamon leaf oil on rumen microbial fermentation using two continuous culture systems. J Dairy Sci.

[11] Newbold CJ, McIntosh FM, Williams P, Losa R, Wallace RJ (2004). Effects of a specific blend of essential oil compounds on rumen fermentation. Anim Feed Sci Technol.

[12] Tokarev V, Lisunova L, Kuz’mina N (2013). Novatan-50 use in lactating cow feed. Russ Agric Sci.

[13] Goering H, Van Soest P (1970). Forage fiber analysis (apparatus reagents, procedures, and some applications). Agriculture Handbook.

[14] Theodorou MK, Williams BA, Dhanoa MS, McAllan AB, France J (1994). A simple gas production method using a pressure transducer to determine the fermentation kinetics of ruminant feeds. Anim Feed Sci Technol.

[15] Pell AN, Schofield P (1993). Computerized monitoring of gas production to measure forage digestion *in vitro*. J Dairy Sci.

[16] Denman SE, McSweeney CS (2006). Development of a real-time PCR assay for monitoring anaerobic fungal and cellulolytic bacterial populations within the rumen. FEMS Microbiol Ecol.

[17] Khafipour E, Li S, Plaizier JC, Krause DO (2009). Rumen microbiome composition determined using two nutritional models of subacute ruminal acidosis. Appl Environ Microbiol.

[18] Livak KJ, Schmittgen TD (2001). Analysis of relative gene expression data using real-time quantitative PCR and the 2^−ΔΔCT^ method. Methods.

[19] Zhan J, Liu M, Su X, Zhan K, Zhang C, Zhao G (2017). Effects of alfalfa flavonoids on the production performance, immune system, and ruminal fermentation of dairy cows. Asian-Australas J Anim Sci.

[20] Wendakoon CN, Sakaguchi M (1995). Inhibition of amino acid decarboxylase activity of Enterobacter aerogenes by active components in spices. J Food Prot.

[21] Castillejos L, Calsamiglia S, Ferret A, Losa R (2007). Effects of dose and adaptation time of a specific blend of essential oil compounds on rumen fermentation. Anim Feed Sci Technol.

[22] Newbold CJ, de la Fuente G, Belanche A, Ramos-Morales E, McEwan NR (2015). The role of ciliate protozoa in the rumen. Front Microbiol.

[23] Koike S, Kobayashi Y (2009). Fibrolytic rumen bacteria: their ecology and functions. Asian-Australas J Anim Sci.

[24] Varel VH, Dehority BA (1989). Ruminal cellulolytic bacteria and protozoa from bison, cattle-bison hybrids, and cattle fed three alfalfa-corn diets. Appl Environ Microbiol.

[25] Wubah DA, Akin DE, Bomeman WS (1993). Biology, fiber-degradation, and enzymology of anaerobic zoosporic fungi. Crit Rev Microbiol.

[26] Yang WZ, Benchaar C, Ametaj BN, Chaves AV, He ML, McAllister TA (2007). Effects of garlic and juniper berry essential oils on ruminal fermentation and on the site and extent of digestion in lactating cows. J Dairy Sci.

[27] Matloup O, El Tawab AMA, Hassan A (2017). Performance of lactating Friesian cows fed a diet supplemented with coriander oil: feed intake, nutrient digestibility, ruminal fermentation, blood chemistry, and milk production. Anim Feed Sci Technol.

[28] Wang R-F, Cao W-W, Cerniglia CE (1997). PCR detection of *Ruminococcus* spp. in human and animal faecal samples. Mol Cell Probes.

[29] Sylvester JT, Karnati SK, Yu Z, Morrison M, Firkins JL (2004). Development of an assay to quantify rumen ciliate protozoal biomass in cows using real-time PCR. J Nutr.

[30] Stevenson DM, Weimer PJ (2007). Dominance of Prevotella and low abundance of classical ruminal bacterial species in the bovine rumen revealed by relative quantification real-time PCR. Appl Microbiol Biotechnol.

